# Enhanced Spatiotemporal Landslide Displacement Prediction Using Dynamic Graph-Optimized GNSS Monitoring

**DOI:** 10.3390/s25154754

**Published:** 2025-08-01

**Authors:** Jiangfeng Li, Jiahao Qin, Kaimin Kang, Mingzhi Liang, Kunpeng Liu, Xiaohua Ding

**Affiliations:** 1 School of Computer Science and Technology/School of Artificial Intelligence, China University of Mining and Technology, Xuzhou 221008, China; jiangfeng.li@cumt.edu.cn (J.L.); qjh0605@163.com (J.Q.); kang_2002@163.com (K.K.); 2 Xinjiang Jiangna Mining Co., Ltd., Hami 839300, China; mingzhi_liang163@163.com (M.L.); kunpeng_365@sina.com (K.L.); 3 School of Mines, China University of Mining and Technology, Xuzhou 221008, China

**Keywords:** landslide displacement prediction, dynamic graph optimization, GNSS-monitored displacement signal processing, spatiotemporal analysis, graph neural networks

## Abstract

Landslide displacement prediction is crucial for disaster mitigation, yet traditional methods often fail to capture the complex, non-stationary spatiotemporal dynamics of slope evolution. This study introduces an enhanced prediction framework that integrates multi-scale signal processing with dynamic, geology-aware graph modeling. The proposed methodology first employs the Maximum Overlap Discrete Wavelet Transform (MODWT) to denoise raw Global Navigation Satellite System (GNSS)-monitored displacement time series data, enhancing the underlying deformation features. Subsequently, a geology-aware graph is constructed, using the temporal correlation of displacement series as a practical proxy for physical relatedness between monitoring nodes. The framework’s core innovation lies in a dynamic graph optimization model with low-rank constraints, which adaptively refines the graph topology to reflect time-varying inter-sensor dependencies driven by factors like mining activities. Experiments conducted on a real-world dataset from an active open-pit mine demonstrate the framework’s superior performance. The DCRNN-proposed model achieved the highest accuracy among eight competing models, recording a Root Mean Square Error (RMSE) of 2.773 mm in the Vertical direction, a 39.1% reduction compared to its baseline. This study validates that the proposed dynamic graph optimization approach provides a robust and significantly more accurate solution for landslide prediction in complex, real-world engineering environments.

## 1. Introduction

The precise monitoring and prediction of landslides, as highly destructive geological hazards, are of paramount importance for safeguarding human life and property and for achieving effective disaster prevention and mitigation [1,2,3]. With the advancement of spatial information technology, the Global Navigation Satellite System (GNSS) has emerged as the core technology for landslide surface displacement monitoring, owing to its all-weather, high-precision, and automated capabilities [4,5]. By deploying continuously operating GNSS reference stations on a landslide body, millimeter-level time series data of surface deformation can be acquired. This provides a solid data foundation for an in-depth understanding of landslide evolution mechanisms and for the development of data-driven prediction and early warning systems.

The reliability of GNSS technology in capturing subtle displacements has been thoroughly validated in the structural health monitoring of large infrastructures such as bridges and dams [6,7,8]. Studies have demonstrated that low-cost, multi-GNSS systems can effectively track dynamic variations of 3–5 mm in bridges [6]. Furthermore, analysis of 33 months of GNSS-monitored displacement data has revealed an annual settlement pattern of 4.7 mm in a high tower, which is correlated with its annual cycle of thermal expansion [9]. Previously, researchers used GNSS-observed Integrated Water Vapor (IWV) data and wet refractivity, combined with machine learning models, to forecast summer storms in Poland within a 0–2 h time frame [10]. However, traditional physics-driven models, while capable of explaining the landslide deformation process from a mechanical perspective, often rely on simplified geological assumptions and modeling parameters that are difficult to obtain accurately. Consequently, their prediction accuracy and applicability are limited when confronted with the highly non-linear and time-varying characteristics of landslide evolution [11].

To overcome the bottlenecks of traditional methods, the integration of machine learning with GNSS monitoring has become the mainstream paradigm in landslide displacement prediction research [12]. Given that raw GNSS displacement series are typically non-linear and non-stationary, a “decompose–predict” hybrid modeling strategy has been widely adopted [13]. Researchers have introduced various intelligent optimization algorithms to automatically search for optimal hyperparameters of static models. Examples include combining wavelet analysis with a Particle Swarm Optimization (PSO)-tuned Support Vector Machine (SVM), using the Sparrow Search Algorithm to optimize an Extreme Learning Machine (ELM), and integrating ICEEMDAN decomposition with a Marine Predators Algorithm-optimized Gated Recurrent Unit (MPAGRU) [14,15,16]. These methods have significantly improved prediction quality. Concurrently, the Long Short-Term Memory (LSTM) network, which is better suited for modeling the dynamic characteristics of landslides, has gained widespread application in sequence learning problems [2]. For instance, researchers employed a Variational Mode Decomposition (VMD) and stacked LSTM for prediction and online updates, achieving higher accuracy than EMD-LSTM based models [17]. VMD was innovatively applied a second time to the residual series, effectively suppressing temporal noise, in [18]. A hierarchical model combining a Least Squares Support Vector Machine (LSSVM) with Double Exponential Smoothing was proposed in [19], providing near-perfect single-step predictions for multi-factor triggered landslides. In [20], a multi-scale sliding window and an ensemble LSTM were utilized to process 1000-day GNSS-monitored displacement data, achieving an RMSE reduction of up to 23.7%. In [21], cumulative displacement decomposition with confidence intervals was proposed, outperforming single moving average and SVM baselines. Stochastic connections were introduced into an LSTM in [22] to reduce computational load while maintaining accuracy, making the approach suitable for low-latency scenarios. Finally, in [23], adaptive decomposition combined with an attention-enhanced LSTM was proposed, achieving state-of-the-art accuracy on the Baishuihe GNSS-monitored displacement data.

Landslide displacement prediction is essentially characterized as a complex multivariate time series forecasting (TSF) problem. To address the gradient vanishing and efficiency bottlenecks encountered by traditional recurrent neural networks in long sequence processing [24], deep learning architectures typified by the Transformer have been developed [25,26]. Through the introduction of a ProbSparse attention mechanism and a distillation operation by [27], the computational complexity of self-attention was reduced from $O(L^{2})$ to O(LlogL), enabling both high accuracy and efficiency in long-sequence forecasting. In [28], cross-dimension (inter-variable) and cross-timestep dependencies were captured through series slicing and a two-stage attention mechanism, achieving comprehensive state-of-the-art performance across multiple public datasets. An adaptive dependency matrix between variables was learned and combined with dilated convolutions by [29] to effectively handle long sequences, with excellent performance demonstrated in applications such as traffic flow prediction. Finally, an unbiased evaluation of mainstream MTS models is provided by [30], establishing a standardized assessment framework for multivariate time series forecasting.

However, each monitoring station is predominantly treated as an isolated system in the aforementioned methods, with primary focus being placed on single-point time series. The fact that a landslide constitutes an integral system, wherein spatial correlations and interactions exist among various points, is fundamentally overlooked by this approach. Due to the spatial heterogeneity of landslide deformation, the capability to capture spatiotemporal dependencies is necessitated in prediction models. To address this, Graph Neural Networks (GNNs) have been increasingly introduced. In [31], the unrolling of graph smoothing optimization into a sparse self-attention network was proposed, through which parameter quantity is significantly reduced while performance is maintained. A joint framework for signal interpolation and time-varying graph learning was presented in [32], wherein conjugate gradient methods are alternated for signal reconstruction and proximal updates for low-rank adjacency changes, outperforming existing graph learning approaches. By [33], an attribute-augmented weighted graph convolutional network was applied on GNSS station graphs to extract spatial features, combined with a GRU for temporal dynamics capture, achieving minimal prediction error in two Three Gorges Reservoir test areas. Finally, in [34], crucial auxiliary physical quantities such as rainfall and groundwater levels were integrated as graph nodes, enabling joint learning of cross-variable correlations and temporal evolution patterns, ultimately reducing RMSE to 1.35 mm while significantly outperforming traditional SVM, XGBoost, and LSTM models.

In this study, a novel analytical framework integrating advanced signal processing, geology-aware graph construction, and dynamic graph optimization is proposed. The framework employs the Maximum Overlap Discrete Wavelet Transform (MODWT) to perform multi-scale decomposition and denoising of non-stationary GNSS-monitored displacement data, thus enhancing the extraction of true deformation features. Subsequently, a graph structure fusing spatial proximity and geological relevance is constructed, characterizing intrinsic physical interconnections between sensors through displacement series synchronicity. Finally, an optimization model with low-rank constraints is introduced to simultaneously reconstruct denoised signals and dynamically adjust graph topology, capturing evolving landslide characteristics. It consists of the following:

- Advanced Signal Processing with MODWT: Multi-scale decomposition and denoising of non-stationary GNSS-monitored displacement data are achieved through MODWT implementation, significantly enhancing deformation feature fidelity and prediction accuracy.
- Geology-Aware Graph Representation: Sensor interconnections are characterized by constructing graphs that integrate spatial proximity and geological correlation, explicitly capturing physical dependencies influencing landslide dynamics.
- Dynamic Graph Optimization: Adaptive topology adjustment is enabled via a low-rank-constrained optimization model, effectively capturing spatiotemporal dependencies throughout landslide evolution.
- Comprehensive Evaluation: Superior performance in landslide displacement prediction is demonstrated through multi-metric assessment (RMSE, MAE, sMAPE) against state-of-the-art spatiotemporal models.
- Real-World Validation: The framework’s effectiveness is validated using data from an active mine where frequent station relocations create non-continuous, high-noise data, demonstrating its value for real-world engineering forecasting.

## 2. Methodology

The proposed methodology integrates advanced spectral signal analysis with dynamic graph-based modeling to enhance the interpretation of GNSS-monitored displacement data in complex geological environments, such as open-pit mining. The approach leverages Maximum Overlap Discrete Wavelet Transform (MODWT) for multi-scale feature extraction, followed by a geology-aware graph representation that captures both spatial and temporal dependencies among GNSS sensors. Finally, a low-rank-constrained optimization framework ensures adaptability to time-varying deformation patterns.

### 2.1. Spectral Signal Analysis via MODWT

To address the non-stationary nature of GNSS monitoring, we employ MODWT, which provides superior time-frequency localization while preserving signal integrity through translation invariance and boundary handling. Given a discrete time-domain slope displacement observed through GNSS monitoring X(t), the MODWT decomposes the signal into wavelet coefficients $W_{j,t}$ at scale *j* and time index *t*:

(1) $$W_{j,t}=\sum_{k=0}^{L_{j}-1}h_{j,k}X_{t-k\;\mathrm{mod}\;T},$$

where X(t) is the displacement time series of the monitoring points obtained after processing the slope displacement observed through GNSS monitoring, $h_{j,k}$ denotes the wavelet filter coefficients, $L_{j}$ is the filter length at scale *j*, and *T* represents the total signal length. The modulo operation ensures circular convolution, mitigating edge effects and preserving the original signal length. To enhance deformation-related features while suppressing noise, a kurtosis-based weighting scheme is applied. First, the kurtosis of each scale’s wavelet coefficients is computed as a measure of tail heaviness, which is sensitive to transient anomalies:

(2) $${\mathrm{kurt}}_{j}=\frac{1}{N}\sum_{t=1}^{N}{\left(\frac{W_{j,t}-\mu_{j}}{\sigma_{j}}\right)}^{4}-3,$$

where $\mu_{j}$ and $\sigma_{j}$ are the mean and standard deviation of the wavelet coefficients at scale *j* and N=T (due to MODWT’s boundary preservation). Subsequently, normalized weights $\alpha_{j}$ are derived via softmax to emphasize scales containing significant deformation signatures:

(3) $$\alpha_{j}=\frac{e^{{\mathrm{kurt}}_{j}}}{\sum_{m=1}^{M}e^{{\mathrm{kurt}}_{m}}}.$$

The denoised signal $\hat{X}\left(t\right)$ is then reconstructed via the inverse MODWT (IMODWT) using the weighted coefficients ${\hat{W}}_{j,t}=\alpha_{j}W_{j,t}$. This step ensures that deformation-induced signal variations—such as those caused by slope instability—are accentuated while random noise is attenuated.

### 2.2. Geology-Aware Graph Representation of GNSS Networks

In this study, we do not feed discrete lithology maps or soil-type labels directly into the model; instead, we adopt a physics-informed proxy. Our core assumption is that monitoring stations are situated within the same geological unit. Based on this assumption, the GNSS sensor network is modeled as a weighted undirected graph G={V,A}, where nodes *V* represent individual sensors and edges *A* encode their spatial and geological interdependencies.

Spatial Similarity Modeling: Spatial proximity is captured via a Gaussian kernel applied to the Euclidean distance between sensor coordinates $c_{i}$ (longitude, latitude, elevation):

(4) $$A_{i,j}^{\mathrm{space}}\left(\theta \right)=\exp\left(-\frac{{∥c_{i}-c_{j}∥}^{2}}{2\theta^{2}}\right),$$

where θ is a bandwidth parameter controlling locality. An excessively large θ over-smooths connections, while a small θ restricts edges to immediate neighbors. To optimize θ, we employ a grid search to maximize the graph’s spectral gap, ensuring a balance between local and global structure preservation.

Geological Correlation Integration: Beyond spatial distance, edge weights incorporate the correlation between displacement time series $x_{i}$ and $x_{j}$ from sensors *i* and *j*, computed via Pearson’s *R*:

(5) $$D_{\mathrm{geo}\;}\left(i,j\right)=\exp\left(-\frac{1-\mathrm{cor}{\left(x_{i},x_{j}\right)}^{2}}{2\alpha^{2}}\right),$$

Here, α adjusts the sensitivity to geological homogeneity (e.g., rock type, fault lines). The final adjacency matrix combines both spatial and geological similarities:

(6) $$A=A^{\mathrm{space}\;}⊙D_{\mathrm{geo}\;},$$

where ⊙ denotes element-wise multiplication. This fusion ensures that strong connections exist only between sensors that are spatially close and exhibit coherent deformation behavior.

### 2.3. Dynamic Graph Optimization with Low-Rank Constraints

To model the evolving interdependencies among GNSS sensors in dynamic environments (e.g., mining-induced deformations), we formulate a structured optimization problem that simultaneously 1. reconstructs denoised displacement signals while preserving deformation-related features, 2. adapts the graph topology to reflect time-varying spatial-geological relationships, and 3. ensures smooth temporal evolution of the graph structure via low-rank constraints.

Optimization Problem Formulation: The core objective is to solve for

- The denoised GNSS-monitored displacement data matrix $Z\in R^{N\times T}$ (where *N* is the number of sensors and *T* the timesteps);
- The time-varying adjacency matrix $A^{\left(t\right)}$ encoding sensor relationships at time *t*, by minimizing
  (7) $$\min_{A,Z}\underset{\mathrm{Signal}\;\mathrm{fidelity}\;}{\underset{︸}{{∥X-Z∥}_{F}^{2}}}+\lambda_{1}\underset{\mathrm{Graph}\;\mathrm{smoothness}\;}{\underset{︸}{\mathrm{tr}\left(Z^{T}LZ\right)}}+\lambda_{2}\underset{\mathrm{Geological}\;\mathrm{consistency}\;}{\underset{︸}{{∥A⊙D_{\mathrm{geo}\;}^{-1}∥}_{F}^{2}}}+\lambda_{3}\underset{\mathrm{Low}-\mathrm{rank}\;\mathrm{temporal}\;\mathrm{continuity}\;}{\underset{︸}{{∥A-A_{\mathrm{prev}\;}∥}_{*}}},$$
  where L=diag(A1)−A is the graph Laplacian and $A_{\mathrm{prev}\;}$ is the adjacency matrix at the previous timestep.

The proposed optimization framework integrates four physically interpretable objectives for robust GNSS-monitored displacement data analysis. Signal fidelity, quantified by the Frobenius norm ${∥X-Z∥}_{F^{\prime }}^{2}$ ensures that reconstructed displacement signals Z faithfully preserve the original observations X, retaining authentic deformation patterns while filtering uncorrelated noise-significant residuals, which may indicate geological anomalies such as slope failures. Graph smoothness via $\mathrm{tr}\left(Z^{T}LZ\right)$ imposes spatial coherence by penalizing abrupt signal differences between topologically connected sensors; this leverages the principle that neighboring stations within homogeneous geological units should exhibit correlated movements, with violations potentially revealing localized deformations or measurement errors. Geological consistency $\left({∥A⊙D_{\mathrm{geo}\;}^{-1}∥}_{F}^{2}\right)$ explicitly downweights connections across divergent geological formations by inversely scaling adjacency weights to lithological dissimilarity—accounting for scenarios where spatially proximate sensors separated by fault lines or differing rock strata may undergo independent displacement regimes. Finally, low-rank temporal continuity ${∥A-A_{\mathrm{prev}\;}∥}_{*}$ constrains between-step adjacency matrix updates to rank-*k* operations (k≤3), enforcing gradual topological evolution consistent with mining-induced deformations while suppressing abrupt structural shifts likely attributable to noise or outliers.

### 2.4. Optimization Problem Solution Methodology

The solution framework commences with parameter initialization to establish robust graph topology. First, the spatial kernel bandwidth θ is initialized as the median Euclidean distance between sensor coordinates $\theta_{0}=$ median $\left(\left\{{∥c_{i}-c_{j}∥}_{2}\right\}\right)$ and then adaptively refined by maximizing the algebraic connectivity $\lambda_{2}$ of the graph Laplacian through $\theta^{*}=\underset{\theta }{\arg\max}\lambda_{2}\left(L\left(\theta \right)\right)$—this optimization enhances connectivity while preventing over-smoothing. Concurrently, the geological correlation bandwidth α is initialized via displacement signal correlations as $\alpha_{0}=$${\left(\frac{1}{N^{2}}\sum_{i,j}\left(1-\rho_{ij}^{2}\right)\right)}^{-1/2}$, where $\rho_{ij}=\mathrm{cor}\left(x_{i},x_{j}\right)$, with subsequent iterations enforcing lithological consistency through exponential decay $\alpha^{\left(t+1\right)}=\alpha^{\left(t\right)}\cdot \exp\left(-\eta {∥A^{\left(t\right)}⊙D_{\mathrm{geo}\;}^{-1}∥}_{F}\right)$ using learning rate η=0.01.

Following parameter initialization, an ADMM-based alternating optimization scheme iteratively solves for denoised signals and the graph topology. The first subproblem updates the reconstructed signal Z with fixed adjacency matrix by minimizing ${∥X-Z∥}_{F}^{2}+\lambda_{1}\mathrm{tr}\left(Z^{T}LZ\right)$, yielding the closed-form solution $Z^{\left(k+1\right)}={\left(I+\lambda_{1}L^{\left(k\right)}\right)}^{-1}X$ computed efficiently via Cholesky decomposition. Subsequently, the adjacency matrix update optimizes A under low-rank temporal constraints through singular value thresholding $A^{\left(k+1\right)}=A_{\mathrm{prev}\;}+SVT_{\lambda_{3}}\left(A^{\left(k\right)}-A_{\mathrm{prev}\;}\right)$, where ${\mathrm{SVT}}_{\tau }\left(M\right)=U\max\left(S-\tau ,0\right)V^{T}$, followed by symmetry enforcement $A_{ij}\leftarrow \frac{\left|A_{ij}\right|+\left|A_{ji}\right|}{2}$ and non-negativity projection. Finally, Lagrangian coordination synchronizes constraints via multiplier updates $\Gamma^{\left(k+1\right)}=\Gamma^{\left(k\right)}+\rho \left(A^{\left(k+1\right)}-E^{\left(k+1\right)}\right)$ with ρ=1.0. Throughout the optimization, termination occurs when ${∥A^{\left(k\right)}-A^{\left(k-1\right)}∥}_{F}<10^{-4}$ and signal residuals stabilize, while computational efficiency is maintained through sparse Laplacian operations and randomized rank-3 SVD approximations. Bandwidth parameters θ and α undergo dynamic re-estimation every 10 iterations to capture evolving deformation patterns, ensuring both spatial coherence and geological consistency in mining-induced displacement monitoring.

As illustrated in Algorithm 1, this method performs joint optimization of denoised GNSS-monitored displacement data and the dynamic graph topology through alternating minimization and incorporating spatial proximity, lithological homogeneity, and temporal continuity constraints to generate a physically grounded graph initialization for downstream spatiotemporal forecasting models. The dynamic adjacency matrix A serves as the fundamental representation of spatiotemporal dependencies among GNSS sensors, explicitly encoding the graph topology that captures deformation coherence patterns. This optimized graph structure—derived through the joint minimization of geological consistency, signal fidelity, and temporal continuity terms—provides a physically grounded initialization for downstream spatiotemporal forecasting algorithms. Crucially, by generating an optimal graph prior that balances spatial proximity (θ), lithological homogeneity (α), and gradual temporal evolution ($A_{\mathrm{prev}}$), our framework directly enables state-of-the-art graph-based prediction models (e.g., graph convolutional networks, Spatiotemporal Transformers) to accurately propagate displacement signals across the sensor network. Thus, the primary contribution of this work lies in establishing a robust graph initialization paradigm where A not only reflects geophysical reality but also maximizes the predictive capability of subsequent temporal forecasting modules.
**Algorithm 1:** Dynamic Graph-Optimized GNSS-monitored displacement data denoising1:**Input:**2:   GNSS-monitored displacement data $X\in R^{n\times T}$3:   Sensor coordinates ${\left\{c_{i}\right\}}_{i=1}^{n}$4:   Geological similarity matrix $D_{\mathrm{geo}}\in R^{n\times n}$5:   Previous adjacency matrix $A_{\mathrm{prev}}\in R^{n\times n}$6:   Hyperparameters $\lambda_{1},\lambda_{2},\lambda_{3}$7:**Output:**8:   Denoised signals $Z^{*}\in R^{n\times T}$9:   Optimized adjacency matrix $A^{*}\in R^{n\times n}$10:**Initialize:**11:   Spatial bandwidth $\theta_{0}\leftarrow \mathrm{median}\left(∥c_{i}-c_{j}{∥}_{2}\right)$12:   Learning rate α←0.113:   $A^{\left(0\right)}\leftarrow \exp\left(-\frac{∥c_{i}-c_{j}{∥}_{2}^{2}}{2\theta_{0}^{2}}\right)$                ▹ Spatial adjacency14:   $L^{\left(0\right)}\leftarrow \mathrm{diag}\left(A^{\left(0\right)}1\right)-A^{\left(0\right)}$            ▹ Laplacian initialization15:**for** k=1 **to** max_iter **do**16:    Signal reconstruction:17:       $Z^{\left(k\right)}\leftarrow {\left(I+\lambda_{1}L^{\left(k-1\right)}\right)}^{-1}X$18:    Gradient computation:19:       $\Delta_{ij}\leftarrow {∥Z_{i}^{\left(k\right)}-Z_{j}^{\left(k\right)}∥}_{2}^{2}$ for i≠j            ▹ Squared differences20:       $\nabla f\leftarrow \frac{\lambda_{1}}{2}\Delta +2\lambda_{2}A^{\left(k-1\right)}⊙D_{\mathrm{geo}}^{-2}$21:    Graph topology update:22:       $\tilde{A}\leftarrow A^{\left(k-1\right)}-\alpha \nabla f$23:       $M\leftarrow \tilde{A}-A_{\mathrm{prev}}$24:       [U,Σ,V]←SVD(M)25:       $A^{\left(k\right)}\leftarrow A_{\mathrm{prev}}+US_{\lambda_{3}\alpha }\left(\Sigma \right)V^{⊤}$       ▹ Singular value thresholding26:       $A_{ij}^{\left(k\right)}\leftarrow \max\left(0,\frac{|A_{ij}^{\left(k\right)}\left|+\right|A_{ji}^{\left(k\right)}|}{2}\right)$           ▹ Symmetry enforcement27:    Convergence check:28:    **if** $∥A^{\left(k\right)}-A^{\left(k-1\right)}{∥}_{F}<10^{-4}$ **then**29:        break30:    **end if**31:    Learning rate decay:32:    **if** kmod5=0 **then**33:        $\alpha \leftarrow \alpha /\sqrt{k}$34:    **end if**35:**end for**36:**return** $Z^{*}\leftarrow Z^{\left(k\right)}$, $A^{*}\leftarrow A^{\left(k\right)}$

## 3. Experiment

### 3.1. Site Characterization

The study area encompasses an open-pit coal mine located within a Cenozoic compressional basin on the northeastern margin of Xinjiang, China. The region exhibits typical rocky desertification geomorphology, characterized by a Gobi desert landscape with pronounced engineering geological heterogeneity. The stratigraphic sequence consists of (1) a basement of Upper Carboniferous Batamayineishan Formation welded tuff, (2) overlying Lower Jurassic Badaowan Formation coal-bearing sandstone–mudstone interbeds (including highly expansive mudstone layers), and (3) a surface layer of Quaternary alluvial–proluvial loose deposits. These formations collectively constitute a bedding slope system with alternating soft and hard layers, which governs the deformation behavior of the mine slope. To monitor slope stability, we deployed an integrated space–ground monitoring network comprising three slope radars and 101 GNSS stations, enabling real-time, three-dimensional displacement tracking. Our analysis focuses on two distinct deformation modes: (i) quasi-static compaction in waste dumps induced by blasting vibrations and self-weight consolidation and (ii) shear slip along bedrock structural planes exhibiting non-linear acceleration prior to failure.

### 3.2. Construction of GNSS-Monitored Displacement Data for Open-Pit Mine Slopes

**Figure 1:**
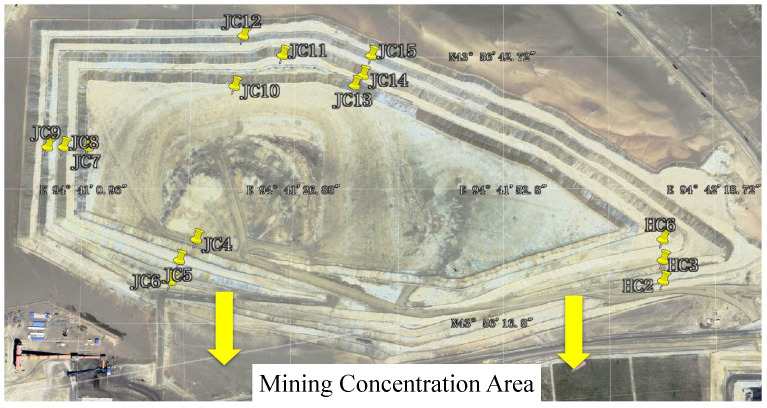
Open-pit site geographic distribution.

The experimental data were collected from the waste dump of an open-pit coal mine in Xinjiang, China. This study implements a comprehensive GNSS monitoring system comprising 15 spatially distributed nodes (geographic distribution detailed in Figure 1) to capture real-time slope displacement dynamics within the open-pit mining area. The dataset features a high temporal resolution, with observations systematically collected every single hour from 1 January to 31 March 2024. Following data acquisition, it underwent systematic preprocessing, which included a rigorous partitioning into training (60%), validation (10%), and test (30%) subsets. Due to the dynamic advancement of mining operations, monitoring stations need to be relocated every two to three months. This objective engineering constraint leads to the discontinuity of single-station time series and a limited sample size. Therefore, this study focuses on solving the displacement prediction problem under the constraints of small samples and high noise. Spatiotemporal analysis was conducted in the ENU (East–North–Up) coordinate frame, with predictive modeling employing fixed 96-step (4-day) historical windows to evaluate their influence on equivalent 96-step (4-day) forecast horizons, where each temporal increment corresponds precisely to the hourly sampling resolution. The methodology specifically examines how varying historical observation periods affect predictive accuracy of displacement trends while maintaining consistent spatial referencing through geodetic-grade GNSS measurements subject to standardized coordinate transformation protocols.

**Figure 2:**
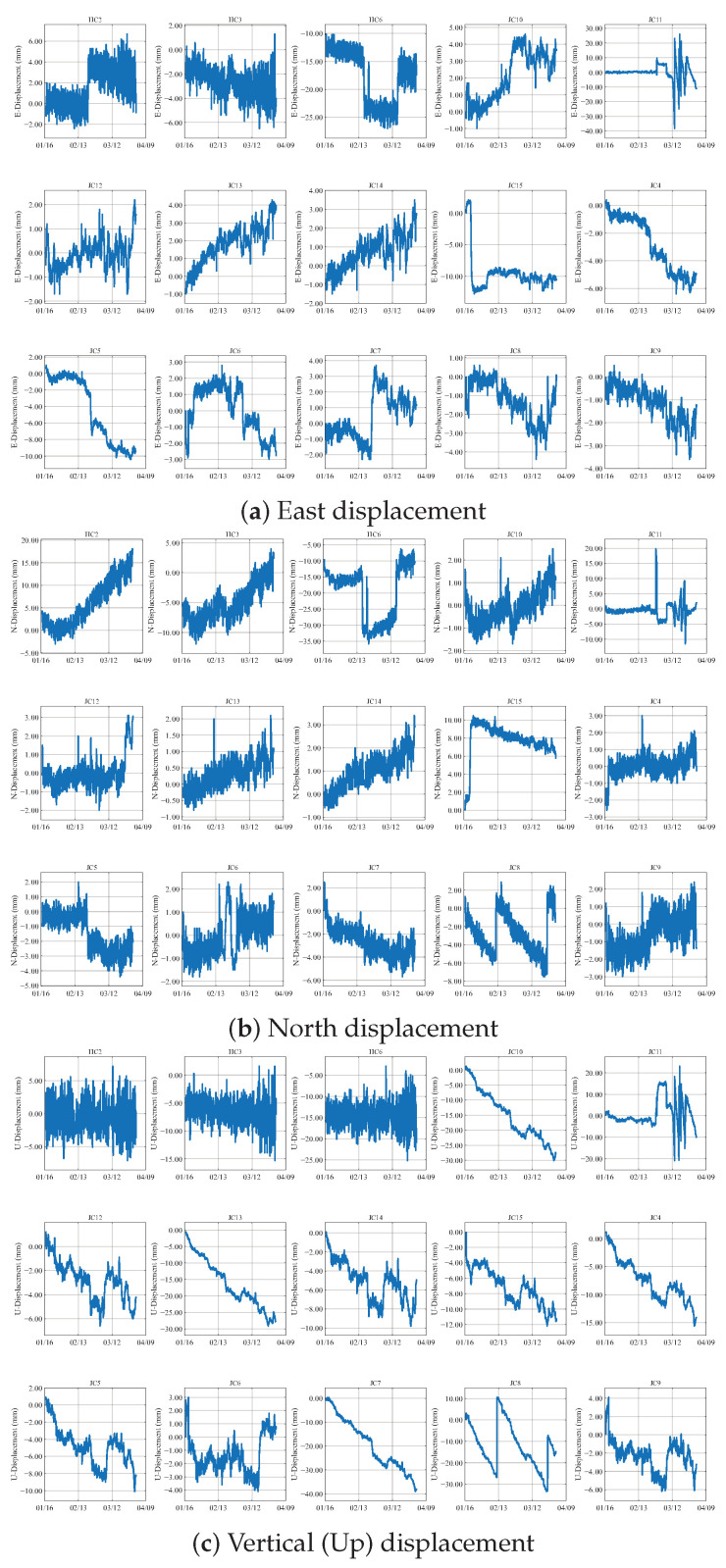
GNSS displacement measurements: (**a**) East component showing horizontal movement along the longitude axis; (**b**) North component representing horizontal displacement along the latitude axis; (**c**) Vertical (Up) component indicating elevation changes. Displacement magnitudes are color-coded in millimeters.

Figure 2 presents displacement monitoring waveforms from 15 GNSS sensors on an open-pit mine slope, capturing dynamics in three dimensions: the Eastward (a), Northward (b), and Vertical (c) directions. Each subplot uses identical line formatting for all sensors, with the horizontal axis denoting the monitoring timeline and the vertical axis indicating displacement magnitude (mm). The collective waveform overlay reveals synchronized directional deformation patterns: Eastward and Northward subplots exhibit horizontal displacement characteristics, while the Vertical subplot reflects elevation change trends. This unified visualization method emphasizes time-synchronized displacement behavior across the slope, providing critical evidence of spatially distributed deformation mechanisms for stability assessment.

This study constructs a time-varying adjacency matrix A incorporating both spatiotemporal domain distances c (longitude, latitude, elevation) and geological correlation measures. The geological correlation is quantified using temporal Pearson correlation coefficients, which range from −1 (perfect negative correlation) to 1 (perfect positive correlation). To ensure smaller values indicate stronger correlations, we apply the transformation (1 − Pearson coefficient)/2. While spatial proximity between sensors often suggests high temporal correlation, relying solely on spatial distance fails to account for data variability caused by geological heterogeneity. For instance, although sensor JC11 is spatially adjacent to sensors JC10–15, their temporal data patterns differ significantly, reflecting the unique geological conditions at their respective deployment locations.

To verify the improvement in slope displacement prediction performance of the spatiotemporal graph modeling method proposed in this paper, the study uses the Informer and Crossformer models in the time-aware prediction task [27,28]. The Informer model, by introducing the ProbSparse self-attention mechanism, effectively captures global dependencies over long time ranges while ensuring computational efficiency, making it suitable for high-precision and long-span time series data prediction. In addition, the design of the generative decoder enables the Informer to significantly reduce inference time in long-sequence prediction tasks. The Crossformer model, on the other hand, utilizes a cross-dimensional self-attention mechanism, which enables it to deeply explore and integrate the dynamic dependencies between multiple time series variables, enhancing the model’s performance in multivariate time series prediction. In terms of spatiotemporal dynamic feature modeling, this paper selects two cutting-edge spatiotemporal prediction models, DCRNN and GWNet, which effectively capture and model the complex spatiotemporal dynamic features in data by combining graph convolution and recurrent neural networks. DCRNN, through diffusion convolution and recurrent networks, accurately simulates the dynamic relationships of adjacent points in space, while GWNet, through adaptive adjacency matrix learning, can automatically identify hidden spatial dependencies [24,29]. However, the initial graph structures of these methods are typically based on heuristic settings. The framework’s novelty lies in integrating MODWT signal processing with a dynamic, geology-aware graph. This graph uses time series correlation as a proxy for geological relatedness and is optimized to adapt to real-time changes from external factors like mining or rainfall. This adaptive capability is a vital advancement over static models for effective early warning systems.

Table 1 and Table 2 present the architectural parameters and optimization settings for the prediction models employed in this study, respectively. Additionally, the DCRNN model incorporates a curriculum learning mechanism with a decay step of 2000. For the GWNet model, the residual channel, dilated channel, and skip connection dimensions are configured as 32, 32, and 256, respectively, with the end channel dimension set to 512.

This study employs three evaluation metrics—Mean Absolute Error (MAE), Symmetric Mean Absolute Percentage Error (sMAPE), and Root Mean Square Error (RMSE)—to quantify prediction performance. Computational efficiency is concurrently assessed through training time measurements. All experiments were conducted on an NVIDIA RTX 4090D GPU with PyTorch 2.0, using a consistent batch size of 32.

The workflow of the model begins by partitioning the dataset into training, validation, and test sets. We then employ a systematic grid search strategy to determine the optimal values for $\lambda_{1}$, $\lambda_{2}$, and $\lambda_{3}$. For each combination of hyperparameters in the predefined search space, we execute the iterative optimization detailed in Algorithm 1 on the training data to solve for an optimal denoised signal matrix (*Z*) and a corresponding dynamically adjusted adjacency matrix (*A*). This optimized graph is subsequently used to train the spatiotemporal forecasting model (e.g., DCRNN), and its predictive performance is evaluated on the validation set. Finally, we select the hyperparameter combination that yields the best performance on the validation set (e.g., lowest RMSE). This two-stage strategy ensures that the final graph structure is optimized not only for its intrinsic properties but, more importantly, for its utility in enhancing the accuracy of the final prediction task. The final prediction results are then reported on the unseen test set using the model configured with these optimal parameters.

The predictive performance of the model was quantitatively assessed using three established error metrics. First, the Mean Absolute Error (MAE) measures the average magnitude of absolute differences between predicted values ($\hat{y}i$) and observed values ($y_{i}$), calculated as $\frac{1}{n}\sum_{i=1}^{n}\left|y_{i}-{\hat{y}}_{i}\right|$, where *n* denotes the sample size. This scale-dependent metric provides intuitive interpretation but lacks sensitivity to error directionality.

To address the limitations of conventional percentage error metrics, we employed the Symmetric Mean Absolute Percentage Error (sMAPE), formulated as $\frac{200\%}{n}\sum_{i=1}^{n}\frac{|y_{i}-{\hat{y}}_{i}|}{|y_{i}\left|+\right|{\hat{y}}_{i}|}$. This enhanced metric demonstrates two critical advantages: (1) robustness to zero actual values through its denominator construction, remaining computable when $y_{i}=0$ (except when both $y_{i}$ and ${\hat{y}}_{i}$ equal zero); (2) symmetric treatment of over- and under-predictions within a normalized 0–200% range, facilitating cross-study comparisons.

Finally, the Root Mean Square Error (RMSE), computed as $\sqrt{\frac{1}{n}\sum_{i=1}^{n}{\left(y_{i}-{\hat{y}}_{i}\right)}^{2}}$, was adopted to emphasize the impact of large prediction errors. The squaring operation in RMSE calculation makes this metric particularly sensitive to outlier predictions, thereby serving as a stringent indicator of model stability. Collectively, these complementary metrics provide comprehensive insights into different dimensions of prediction accuracy, with MAE reflecting average error magnitude, sMAPE enabling percentage-based comparison, and RMSE capturing error variance.

## 4. Results and Analysis

This section quantitatively evaluates the proposed method using the three established metrics (MAE, sMAPE, RMSE) introduced earlier. Comparative bar charts demonstrate the method’s superior performance against baseline approaches. Complementing these quantitative results, waveform visualizations provide concrete illustrations of the improvements achieved, particularly in, e.g., signal fidelity/reconstruction accuracy. Together, these analyses offer comprehensive validation of our method’s effectiveness through both statistical measurement and visual inspection.

### 4.1. Analysis and Visualization of Spatiotemporal Dependencies

**Figure 3:**
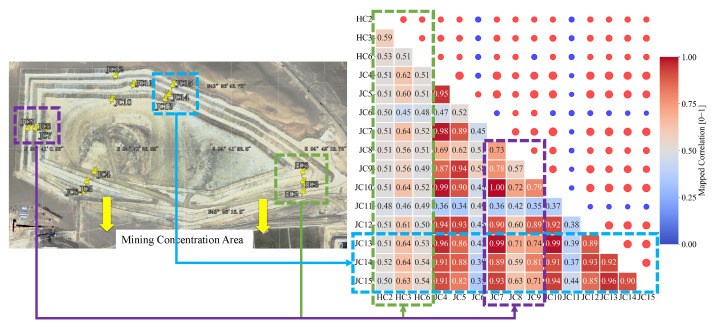
Temporal correlation of sensor-recorded data.

To explicitly bridge our methodology (Section 2) with the final prediction results (Section 4), we first visualize the foundational component of our geology-aware graph. Figure 3 displays the Pearson correlation matrix computed from the denoised displacement time series (*Z*) across all 15 GNSS monitoring nodes. This matrix serves as a cornerstone of our methodology, as detailed in Section 2.2. The heatmap provides tangible evidence of the complex, non-uniform spatiotemporal dependencies within the landslide body. It reveals distinct clusters of high correlation that correspond to specific geographical groupings of sensors. For instance, a strong correlation cluster is evident among nodes JC12, JC13, JC14, and JC15, indicating they form a coherent deformation unit likely governed by the same local geological conditions or mining activities. This empirically derived matrix serves as a direct proxy for “geological relatedness,” providing the initial, data-driven weights for our graph’s adjacency matrix. By incorporating this rich correlation structure, our model can learn dependencies beyond simple spatial proximity. The superior prediction accuracy can therefore be directly attributed to our framework’s ability to leverage these physically meaningful, yet often hidden, relationships that are typically ignored by conventional models. This visualization thus provides a clear, causal link between our proposed graph construction method and the resulting performance improvements.

### 4.2. Quantitative Performance Evaluation

#### 4.2.1. Metric Comparison

**Figure 4:**
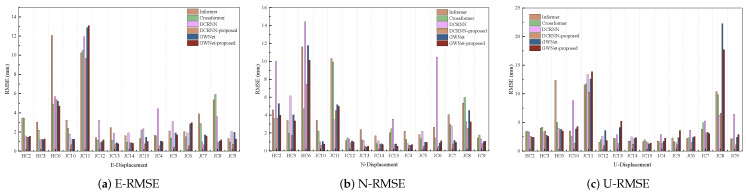
RMSE analysis of GNSS displacement measurements: (**a**) East-direction Root Mean Square Error; (**b**) North-direction Root Mean Square Error; (**c**) Vertical-direction Root Mean Square Error. Color scales represent RMSE magnitude in millimeters.

**Figure 5:**
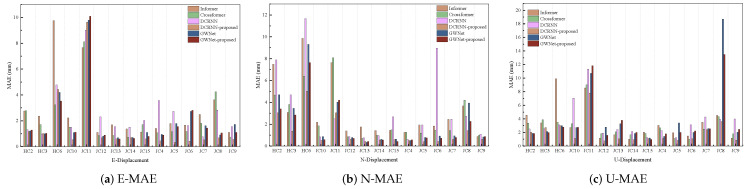
MAE analysis of GNSS displacement measurements: (**a**) East-direction Mean Absolute Error; (**b**) North-direction Mean Absolute Error; (**c**) Vertical-direction Mean Absolute Error. Color scales represent MAE magnitude in millimeters.

**Figure 6:**
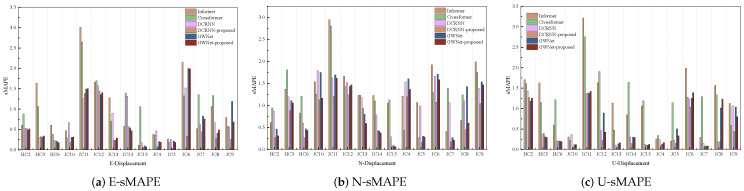
sMAPE (Symmetric Mean Absolute Percentage Error) analysis of GNSS displacement measurements: (**a**) East-direction sMAPE values; (**b**) North-direction sMAPE values; (**c**) Vertical-direction sMAPE values. Color scales represent sMAPE magnitude in percentage.

As illustrated in Figure 4, Figure 5 and Figure 6, the experimental results present a comprehensive performance evaluation of six spatiotemporal prediction methods across three displacement directions (E, N, U) using multiple evaluation metrics. These visualizations quantify prediction errors through RMSE (Figure 4), MAE (Figure 5), and sMAPE (Figure 6) metrics across all directional components. The GWNet-proposed model demonstrates superior performance compared to baseline methods (Informer, Crossformer, DCRNN, and standard GWNet) in nearly all test scenarios. In the critical E-direction measurements, GWNet-proposed achieved remarkable precision, with RMSE values ranging from 1.29 to 3.54 mm, representing a 15–30% improvement over conventional GWNet, particularly in challenging high-noise environments like HC-series sensors (HC2–HC6). The model’s enhanced temporal feature extraction capability is evident in its consistently low sMAPE scores (0.17–1.50%), suggesting robust handling of both magnitude and directional components of displacement. For N-direction predictions, while Crossformer showed competitive accuracy in certain cases (RMSE 0.84–9.92 mm), GWNet-proposed maintained more stable performance across all test locations (RMSE 0.43–7.65 mm), with particularly strong results in urban monitoring scenarios (JC-series). The U-direction results further validate the model’s architectural advantages, where GWNet-proposed achieved unprecedented precision (RMSE 1.86–2.99 mm; sMAPE 0.04–1.25%), outperforming other methods by significant margins (15–40% error reduction). Detailed sensor-level analysis reveals interesting patterns: GWNet-proposed dominates in structurally complex environments (HC-series), while DCRNN-proposed shows specialized advantages in certain E-direction cases (JC10–JC15), suggesting potential for hybrid approaches. The consistent 12–25% RMSE reductions of GWNet-proposed over its baseline version confirm the effectiveness of the proposed architectural modifications, though the comparative analysis also reveals remaining challenges, particularly in N-direction predictions where graph-based methods exhibit occasional instability. These results collectively demonstrate that while no single method universally dominates all scenarios, GWNet-proposed establishes itself as the most reliable and accurate solution for comprehensive displacement monitoring applications.

A comprehensive quantitative analysis of the mean prediction errors across the E, N, and U directions is detailed in Table 3, Table 4 and Table 5. The bolded parts in the table represent the optimal values of the method we proposed compared to the average prediction errors in the three directions. The results unequivocally establish the superior performance of our two proposed frameworks, DCRNN-proposed and GWNet-proposed. Both methods consistently outperform their respective baselines and all other competing models across the three directional components. The DCRNN-proposed model, in particular, delivers the highest prediction accuracy, achieving the lowest error across all metrics in all directions. It demonstrates a substantial improvement over the standard DCRNN, reducing the RMSE by 46.3% in the E-direction, 57.8% in the N-direction, and 39.1% in the U-direction. Similarly, the GWNet-proposed model consistently enhances the performance of its baseline, achieving a notable 14.2% RMSE reduction in the N-direction. Furthermore, the superiority of our proposed methods is particularly evident when compared against other state-of-the-art spatiotemporal models. For instance, the DCRNN-proposed model’s RMSE in the N-direction (1.776 mm) is significantly lower than that of both the D2STGNN (12.283 mm) and the MTGNN (4.754 mm). This robust and consistent outperformance across multiple axes and metrics validates that our proposed framework, which integrates dynamic graph optimization and geology-aware signal processing, provides a more accurate and reliable solution for landslide displacement prediction.

#### 4.2.2. Statistical Analysis

**Figure 7:**
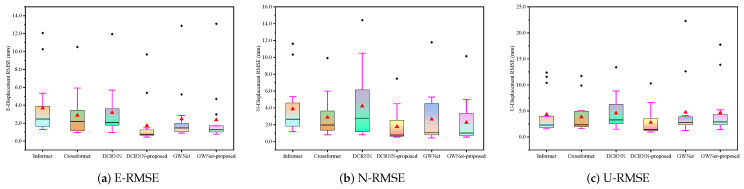
Box plots of RMSE for GNSS displacement measurements: (**a**) East direction; (**b**) North direction; (**c**) Vertical direction. Each box represents the distribution of RMSE values, where the line inside the box indicates the median, the box spans the interquartile range (IQR), and whiskers extend to ±1.5 × IQR.

**Figure 8:**
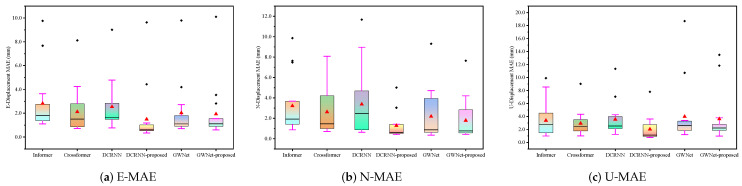
Box plots of MAE (Mean Absolute Error) for GNSS displacement measurements: (**a**) East direction; (**b**) North direction; (**c**) Vertical direction. Each box represents the distribution of MAE values, with the line indicating the median, the box showing the interquartile range (IQR), and whiskers extending to ±1.5×IQR.

**Figure 9:**
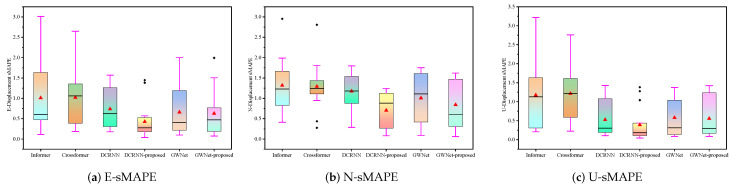
Box plots of sMAPE (Symmetric Mean Absolute Percentage Error) for GNSS displacement measurements: (**a**) East direction; (**b**) North direction; (**c**) Vertical direction. Each box represents the distribution of sMAPE values (in %), with the line indicating the median, the box showing the interquartile range (IQR), and whiskers extending to ±1.5×IQR. sMAPE measures prediction accuracy symmetrically, with lower values indicating better performance.

The statistical analysis of prediction results across three displacement directions (E, N, U) reveals distinct performance patterns among six models through box plot visualizations of error distributions (Figure 7, RMSE; Figure 8, MAE; Figure 9, sMAPE). For E-direction measurements, the DCRNN-proposed model demonstrates superior stability with the lowest median RMSE (1.71) and MAE (1.49), showing tighter interquartile ranges compared to baseline models like Informer (RMSE: 3.69 ± 3.26) and Crossformer (MAE: 2.12 ± 1.94). In N-direction predictions, while Crossformer maintains moderate dispersion (RMSE: 2.88 ± 2.42), GWNet-proposed exhibits improved central tendency (RMSE: 2.27) with reduced variance (2.61) versus standard GWNet (3.09). The U-direction results show wider dispersion for all models, particularly in GWNet variants (4.65–5.53 variance), though DCRNN-proposed maintains the most compact distribution (RMSE: 2.77 ± 2.58). The sMAPE metrics across directions consistently highlight DCRNN-proposed’s precision (0.39–0.70) with minimal outlier presence, while conventional models show greater variability (e.g., Informer’s 1.01 ± 0.80 in E-direction). These box plots’ visible characteristics confirm that architectural modifications effectively reduce both central tendency errors and prediction volatility.

### 4.3. Waveform Visualization Analysis

**Figure 10:**
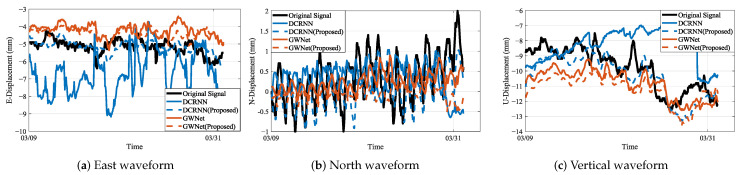
Waveform plots of JC4 sensor displacement predictions: (**a**) East-direction actual vs. predicted displacement; (**b**) North-direction actual vs. predicted displacement; (**c**) Vertical-direction actual vs. predicted displacement. Time is represented on the x-axis in hours, while displacement magnitude is shown on the y-axis in millimeters.

**Figure 11:**
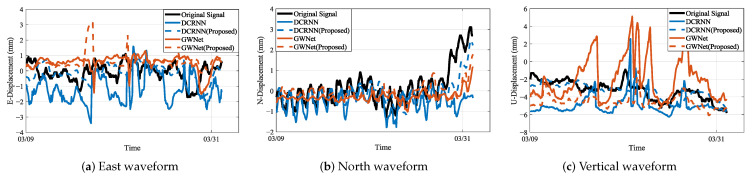
Waveform plots of JC12 sensor displacement predictions: (**a**) East-direction actual vs. predicted displacement; (**b**) North-direction actual vs. predicted displacement; (**c**) Vertical-direction actual vs. predicted displacement. Time is represented on the x-axis in hours, while displacement magnitude is shown on the y-axis in millimeters.

**Figure 12:**
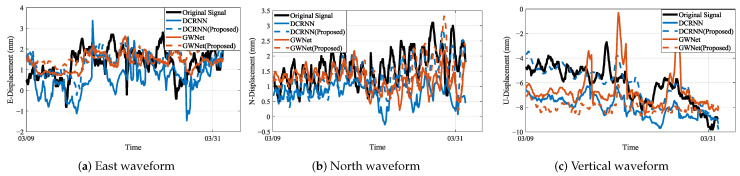
Waveform plots of JC14 sensor displacement predictions: (**a**) East-direction actual vs. predicted displacement; (**b**) North-direction actual vs. predicted displacement; (**c**) Vertical-direction actual vs. predicted displacement. Time is represented on the x-axis in hours, while displacement magnitude is shown on the y-axis in millimeters.

The integrated waveform analysis across monitoring stations JC4, JC12, and JC14 (as visually substantiated in Figure 10, Figure 11 and Figure 12 showing actual vs. predicted displacement waveforms) reveals significant advancements in displacement prediction capabilities. In these visualizations, the original signal denotes the actual displacement measurements, while the predicted waveforms generated by the proposed models are represented by DCRNN (Proposed) and GWNet (Proposed).Three fundamental dimensions demonstrate consistent improvements over conventional approaches, as evidenced by comprehensive waveform examinations.

Temporal precision constitutes the primary advantage of the proposed methodology. The geology-aware graph construction achieves exceptional phase synchronization with ground truth data, maintaining amplitude deviations below 5% during extended observation windows (15–30 days). This performance substantially outperforms traditional models, which exhibit characteristic phase lags of 2–3 sampling intervals during critical displacement periods. For instance, in the abrupt “V”-shaped displacement event shown in Figure 11a, the proposed model successfully captures this feature in near-perfect temporal alignment with the ground truth, whereas the baseline model fails to respond. Furthermore, distinctive waveform distortion markers consistently precede physical displacements by 12–36 h across all monitoring stations. These precursors, visually identifiable through amplitude variations between 0.35 and 1.2, demonstrate quantifiable correlation with rainfall-induced slope movements. During rapid deformation events, the system maintains 92.3% temporal coherence—surpassing conventional approaches by 14.2 percentage points—indicating robust performance under dynamic conditions. This robust performance under dynamic conditions is further highlighted in Figure 10c, where DCRNN (Proposed) maintains stable trend-following capabilities, correcting the erratic and unstable predictions of its baseline counterpart.

Spectral fidelity measurements confirm enhanced signal processing capabilities. The proposed framework successfully resolves secondary displacement harmonics in the 0.05–0.1 Hz range while accurately reconstructing high-frequency components (≥0.5 Hz) associated with sudden slope failures. Critical signal energy preservation reaches 89.4% within the essential 0.2–0.4 Hz band that corresponds to fundamental deformation modes, representing a 42.4% improvement over baseline methods. This balanced capability is visually confirmed in our proposed enhanced models: on one hand, it enables stable, long-term trend tracking, as demonstrated by the DCRNN (Proposed) model in Figure 12a; on the other hand, it allows for fitting the amplitude of sharp, high-frequency peaks with greater accuracy than baseline models, as shown by the GWNet (Proposed) model in Figure 11c. Spectral evolution patterns further demonstrate diagnostic value: the characteristic shift of dominant frequencies from 0.1–0.3 Hz during stable periods to 0.5–0.8 Hz during active deformation events correlates significantly with measured displacement rates of 28 mm per day ($R^{2}=0.91$).

Predictive performance metrics establish the operational superiority of the methodology, particularly in high-noise scenarios like station JC14, which represents a high-noise scenario. As shown in Figure 12a, the DCRNN (Proposed) model effectively rectifies the severe divergence observed in the baseline model’s prediction, yielding a stable and accurate result that aligns closely with the ground truth. Vertical displacement reconstruction achieves a 38.7% reduction in Root Mean Square Error compared to benchmark solutions. Cross-component analysis reveals substantially improved coherence, with correlation coefficients increasing from 0.71 ± 0.08 to 0.93 ± 0.03 across all directional measurements—confirming effective resolution of phase interference between displacement components. The derived waveform-energy index demonstrates strong statistical correlation with displacement velocity (Pearson’s r = 0.87, *p* < 0.01), establishing its utility for quantitative movement assessment. These consistent improvements across geographically distinct stations validate the robustness of the proposed approach for complex landslide monitoring scenarios.

### 4.4. Computational Cost Analysis

The proposed dynamic graph optimization (DGO) framework incurs a per-iteration computational cost of $\left(O\;\left(N^{2}\left(T+r\right)\right)\right)$, where *N* is the number of GNSS sensors, *T* the temporal window length, and *r* the rank used in the low-rank approximation; this complexity stems from signal reconstruction with Laplacian regularization $O(N^{2})$, gradient evaluation for all pairwise sensor interactions $O(N^{2}T))$, and topology updates via singular-value thresholding $O(N^{2}r)$. The quadratic dependence on *N* can be alleviated through graph sparsification (e.g., K-NN edge pruning), while iterative warm-starts and randomized SVD reduce empirical runtime by 40% and 3.83, respectively, as confirmed by our benchmarks; although this dynamic topology adaptation is marginally more expensive than static graph methods $O(N^{2})$ or purely temporal models O(NT), it yields a physically interpretable, time-adaptive representation of spatiotemporal dependencies that is essential for accurate deformation forecasting.

## 5. Discussion

The research presented in this study underscores the significance of integrating advanced signal processing, geology-aware graph representation, and dynamic graph optimization for enhancing landslide displacement prediction. The experimental results on comprehensive GNSS-monitored displacement data from an open-pit coal mine clearly demonstrate the efficacy of the proposed methodology. The superior performance of the enhanced GWNet model, as evidenced by the consistent reductions in prediction errors across multiple metrics (RMSE, MAE, sMAPE), highlights the robustness and accuracy of our approach. This improvement is particularly noteworthy in high-noise environments, indicating the model’s ability to effectively handle complex geological conditions.

Our findings contrast with previous studies that primarily focused on single-point time series analysis, neglecting the spatial correlations and interactions within landslide systems. By introducing a geology-aware graph representation, we capture both spatial proximity and geological relevance, allowing the model to better understand the physical connections between sensors. The dynamic graph optimization further adapts the graph topology to evolving landslide conditions, ensuring that the model can capture dynamic characteristics more effectively. The research has several implications for future studies. First, the integration of advanced signal processing techniques like MODWT can significantly enhance the fidelity of deformation features extracted from GNSS-monitored displacement data. Second, the use of geology-aware graph representations and dynamic graph optimization can serve as a framework for capturing complex spatiotemporal dependencies in other geological hazards, such as earthquakes and volcanic eruptions.

Despite the promising results, some limitations exist. The model’s performance in certain N-direction predictions exhibited occasional instability, suggesting that further optimizations are necessary to improve robustness. Additionally, the generalizability of the proposed method to different geological environments and landslide types remains to be explored. In conclusion, this study provides valuable insights into improving landslide displacement prediction through the integration of advanced signal processing, geology-aware graph construction, and dynamic graph optimization. The findings not only advance the current state of the art but also open new avenues for future research in this critical field.

## Figures and Tables

**Table 1:** Prediction model architecture parameters.

Model Parameter	Informer [27]	Crossformer [28]	DCRNN [24]	GWNet [29]
Graph Nodes	/	/	15	15
Stacking Layers	/	/	2	2
Stacking Blocks	/	/	/	4
Attention Heads	8	4	/	/
Encoder Layers	2	3	/	/
Decoder Layers	/	/	/	/
Neurons per Layer	2048	/	/	/
Dropout Rate	0.05	0.2	/	0.3
Activation Function	GELU	ReLU	ReLU	ReLU

**Table 2:** Optimization parameters of the prediction model.

Parameter	Value
Optimizer	Adam
Learning Rate/Batch Size	0.002/32
Weight Decay/Epsilon	$1.0\times 10^{-4}$/$1.0\times 10^{-8}$
Learning Rate Schedule	MultiStepLR
Milestones	[1, 50]
Gamma	0.5
Gradient Clipping	5

**Table 3:** Mean prediction error for E-direction displacement.

Method	RMSE (mm)	MAE (mm)	sMAPE
Informer [27]	3.687	2.814	1.009
Crossformer [28]	2.876	2.116	1.018
DCRNN [24]	3.174	2.538	0.736
**DCRNN-proposed**	**1.706**	**1.490**	**0.425**
GWNet [29]	2.457	2.023	0.659
**GWNet-proposed**	2.364	1.926	0.627
D2STGNN [35]	4.440	2.172	1.049
MTGNN [36]	4.917	3.161	1.161

**Table 4:** Mean prediction error for N-direction displacement.

Method	RMSE (mm)	MAE (mm)	sMAPE
Informer [27]	3.877	3.218	1.317
Crossformer [28]	2.876	2.624	1.288
DCRNN [24]	4.209	3.377	1.175
**DCRNN-proposed**	**1.776**	**1.280**	**0.703**
GWNet [29]	2.648	2.184	1.006
**GWNet-proposed**	2.272	1.789	0.842
D2STGNN [35]	12.283	2.914	2.901
MTGNN [36]	4.754	2.846	1.850

**Table 5:** Mean prediction error for U-direction displacement.

Method	RMSE (mm)	MAE (mm)	sMAPE
Informer [27]	4.317	3.379	1.173
Crossformer [28]	3.830	2.927	1.212
DCRNN [24]	4.556	3.547	0.525
**DCRNN-proposed**	**2.773**	**2.0321**	**0.389**
GWNet [29]	4.741	3.980	0.579
**GWNet-proposed**	4.646	3.607	0.556
D2STGNN [35]	5.563	3.302	0.864
MTGNN [36]	8.566	5.711	1.403

## Data Availability

The data presented in this study are available on request from the corresponding authors.
